# Genome-wide identification of the E-class gene family in wheat: evolution, expression, and interaction

**DOI:** 10.3389/fpls.2024.1419437

**Published:** 2024-09-03

**Authors:** Xionghui Bai, Pengfei Qiao, Hanxiao Liu, Yuping Shang, Jie Guo, Keli Dai

**Affiliations:** College of Agronomy, Key Laboratory of Sustainable Dryland Agriculture (Co-construction by Ministry and Province), Ministry of Agriculture and Rural Affairs, Shanxi Agricultural University, Jinzhong, China

**Keywords:** wheat, E-class genes, evolution, flower, interaction

## Abstract

**Introduction:**

Wheat (*Triticum aestivum* L.) is among themost important crop worldwide. Given a growing population and changing climate, enhancing wheat yield is of great importance. Yield is closely associated with flower and spike development, and E-class genes play important roles in the flower and kernel development of plants. Currently, the absence of systematic analysis on the E gene family hinders our comprehension of their roles in plant growth and development.

**Methods:**

Identify E-class genes based on homologous sequence searches. Analyze the identified E-class genes through a series of gene family analyses. Determine the expression levels of wheat E-class genes by searching public databases. Validate the functions of these genes by transforming them into *Arabidopsis*. Finally, determine the interactions between the genes through yeast two-hybrid experiments.

**Results:**

Fifteen E-class genes (TaEs) were identified in common wheat. Nine E-class genes were detected in five ancestral/closely related species, including one in *Aegilops tauschii* (AtE), one in *T. Urartu* (TuEs), two in *T. turgidum* (TtEs), two in *T. dicoccoides* (TdEs), and three in *T. spelta* (TsEs). The 24 E-class genes were classified into three subgroups using a phylogenetic approach. All genes were highly expressed in spikes, and most were only highly expressed at the floret meristem stage. The effects of *TaSEP5-A* on flowering and growth cycles were confirmed in homologous mutants and transgenic *Arabidopsis thaliana*. The E-class genes were able to regulate the growth cycle of *Arabidopsis*. Finally, we confirmed the interactions between *TaSEP5-A* and other wheat E-class genes based on yeast two-hybrid assays.

**Discussion:**

Our findings provide information regarding the E-class genes in wheat and will potentially promote the application of these genes in wheat improvement.

## 1 Introduction

Wheat (*Triticum aestivum* L.) is a vital cereal crop cultivated worldwide. It serves as a major source of daily caloric intake for over one-fifth of the global population and accounts for approximately 28% of global cereal production (Tu and Li, 2020). The projected global population will reach 9.9 billion by 2050 (Population Reference Bureau, 2020; https://www.stats.gov.cn/). An annual growth rate of 2% in wheat production is required to meet the increasing demand. Consequently, a comprehensive understanding of the genes associated with functional traits assumes critical significance in the molecular breeding of wheat, presenting substantial implications for yield and quality enhancement.

Flower development has been the focus of extensive research, particularly in dicotyledonous plants such as *Arabidopsis thaliana* and *Plantago asiatica* (Jack, 2004). Those investigations have contributed to our overall comprehension of floral organ development in higher plants, leading to the proposition of the ABCDE model, which delineates the classification of plant floral organs based on the functions of A-, B-, C-, D-, and E-class genes (Zahn et al., 2006). According to the ABCDE model, the first floral whorl is specified by the A- and E-class genes, governing sepal development. In the subsequent whorl, petal development is regulated by the A-, B-, and E-class genes. The third whorl is marked by the actions of B-, C-, and E-class genes, determining stamen formation. In the fourth whorl, carpel differentiation is controlled by the C- and E-class genes. Finally, the D- and E-class genes are responsible for ovule development within the pistil.

Notably, the E-class genes play crucial roles in the morphological construction of all floral organs. In *Arabidopsis*, these genes encompass four members: *SEPALLATA1* (*SEP1*), *SEP2*, *SEP3*, and *SEP4*, which display partially redundant functions in the development of petals, stamens, and carpels (Ditta et al., 2005; Honma and Goto, 2001; Pelaz et al., 2000). Through the course of evolution, the diversification of the E-class genes has contributed to substantial changes in floral morphology among terrestrial plants (Irish and Litt, 2005). Studies on monocotyledonous plants like rice (*Oryza sativa* L.), similar to those on common wheat, have categorized the E-class genes into *SEP* and *OsMADS1* clades. The simultaneous knockout of all the four rice *SEP* genes resulted in the homologous transformation of all floral organs, except for the lemmas, giving rise to leaf-like structures. Conversely, the *OsMADS1* mutant phenotype in rice exhibited leaf-like lemmas and paleas (Agrawal et al., 2005; Malcomber and Kellogg, 2004). Moreover, duplication of the E-class genes potentially contributes to the increased complexity of regulatory mechanisms underlying floral organ formation (Cui et al., 2010). Therefore, the identification and functional analysis of the E-class genes in crops is essential for improving our understanding of floral development processes in these plants, thereby enhancing crop yields.

Spike development plays a crucial role in determining wheat yield (Kellogg et al., 2001). A wheat spike is composed of multiple florets (Kong et al., 2022). In common wheat, the process of inflorescence development occurs in distinct stages. Initially, the inflorescence meristem forms a specialized structure known as the spikelet meristem, which is found in the axils of leaves. Subsequently, the spikelet meristem differentiates into floret meristems, giving rise to floral organs (Sakuma et al., 2019). Each spike typically contains six to eight florets, with some located at the top. Internally, each floret comprises protective structures called the lemma and palea (Liu et al., 2021; Park et al., 2014). The lemma is derived from the axillary meristem, and the palea develops from the primordial leaf formed by the axillary meristem. A wheat flower consists of a pistil, three stamens, and two vestigial leaf-like structures called lodicules. The pistil is composed of three fused carpels, while the stamens consist of filaments and anthers. During flowering, the lodicules swell, which causes the lemma and pedicel to separate, facilitating the release of pollen from anthers onto the receptive stigma (Dobrovolskaya et al., 2015; Li et al., 2019). Overall, spike development in common wheat is closely linked to the development of floral organs, including the lemma, palea, stamens, and pistil (Ikeda et al., 2007; Ikeda-Kawakatsu et al., 2012). The proper establishment of these floral structures is regulated by the E-class genes, which play a critical role in spike and floral development (Yoshida et al., 2013).

Common wheat is an allohexaploid plant species with a genetic makeup of 2n = 6x = 42. It consists of three homoeologous sub-genomes (A, B, and D) (Dubcovsky and Dvorak, 2007; Lev-Yadun et al., 2000). This characteristic makes it an excellent model for studying sub-functionalization and polyploidy in plants (Mcfadden and Sears, 1946). Cultivated wheat as we know it today emerged approximately 8,500–9,000 years ago through hybridization events involving the domesticated tetraploid ancestors *T. turgidum* (AABB) and *T. dicoccoides* (AABB) and the diploid donor of the D sub-genome, *Aegilops tauschii* (DD) (Jia et al., 2013; Luo et al., 2017; Marcussen et al., 2014; Shewry and Hey, 2015). The identity of one diploid donor, *T. urartu* (A sub-genome), has been confirmed in one of the sub-genomes of the tetraploid ancestor (Avni et al., 2017; Maccaferri et al., 2019; Zhao et al., 2017). However, the exact origin of the B sub-genome, which is the cytoplasmic donor of common wheat, remains uncertain. It is either extinct, yet to be discovered, or part of a polyphyletic origin (Levy and Feldman, 2022; Li et al., 2021; Tsunewaki et al., 2019). Common wheat quickly spread globally from its origin in the Fertile Crescent, becoming a staple food for humans (Dubcovsky and Dvorak, 2007; Lémin et al., 2019; Mayrose et al., 2011; Mirzaghaderi and Mason, 2017). This expansion may have been facilitated by allopolyploidy, which promotes genetic variation, novel trait acquisition, intergenomic interactions, and mutational buffering, thus satisfying the yield and quality requirements of human agriculture (Murat et al., 2017; Rice et al., 2019; Salse et al., 2008; Wang et al., 2015).

Despite significant advancements in plant genomics, there remains a crucial research gap in understanding the comprehensive evolutionary and functional dynamics of E-class genes in wheat (Kong et al., 2022). In this study, we utilized machine learning algorithms to optimize the classification and functional prediction of genes (Homology Analysis, Domain Analysis, Gene Co-expression Network Analysis, etc.). Due to the highly specific functions of these genes, it is not possible to identify them using a single-domain Hidden Markov Model. Therefore, we employed a homologous gene search approach to identify these genes. We identified 15 E-class genes (TaEs) in common wheat, the result is consistent with previous research findings (Kong et al., 2022), but our approach treats E-class genes as a cohesive gene family, aimed at uncovering novel insights, one in *A. tauschii* (AtE), one in *T. urartu* (TuE), two in *T. turgidum* (TtEs), two in *T. dicoccoides* (TdEs), and three in *T. spelta* (TsEs). We conducted a comprehensive investigation into the evolutionary and structural characteristics of the TaEs through phylogenetic, gene domain and conserved motif, chromosomal distribution, and collinearity analyses. Furthermore, we determined expression patterns and functions of the TaEs using RNA-seq data and *cis*-element analysis. We performed *Arabidopsis* transformation experiments to validate the functions of the target genes. Finally, we confirmed the interactions among the E-class genes using a yeast two-hybrid assay. Our study fills this critical research gap by conducting a thorough gene family analysis of the E genes, providing novel insights into their evolutionary dynamics within wheat. By elucidating the evolutionary journey of E genes, we offer a unique perspective on the evolutionary history of wheat, contributing significantly to the fields of plant genetics and evolution research.

## 2 Materials and methods

### 2.1 Identification of the E-class genes in common wheat and its relatives

The protein sequences for the E-class genes of *Arabidopsis* and proteomic data of common wheat were obtained from the Ensembl Plant database (https://plants.ensembl.org/index.html). They were used to perform a BLASTP (Basic Local Alignment Search Tool Protein) search against the common wheat protein database using an Expect value (e value) of 10^−5^ (Lei et al., 2021). The same method was employed to determine the E-class genes of ancestral/closely related species of common wheat, including *A. tauschii, T. dicoccoides*, *T. spelta*, *T. turgidum*, and *T. urartu*). Values for molecular weight (Mw), isoelectric point (pI), instability index (II), aliphatic index (AI), and grand average of hydropathicity (GRAVY) of the identified E-class proteins were calculated using the ExPASy online software (http://web.expasy.org/protparam/) (Wilkins et al., 1999), whereas their subcellular localization was predicted using the Cell Ploc 2.0 online tool (http://www.csbio.sjtu.edu.cn) (Yang, 2005). Homologous gene pairs were identified based on chromosomal location and sequence similarity. Molecular selection effects were assessed based on calculations of the non-synonymous substitution/synonymous substitution (dN/dS) values using the codeml program in the PAML package (Guo et al., 2017). The genes with dN and dS values of 0 were eliminated, as this may be attributable to sequence saturation or sequence mismatches (Barberis et al., 2021). Genes with dN/dS values >1 were designated positively selected genes (PSGs), and those with dN/dS values <1 were considered negatively selected genes (NSGs).

### 2.2 Phylogenetic, motif, and domain analyses

To gain further understanding of the evolutionary relationships among the E-class genes, we compared the genes in *Arabidopsis*, rice, common wheat and its ancestral/closely related species (Larkin et al., 2007). Full-length alignment of the E-class gene sequences was performed using MUSCLE, and neighbor-joining (NJ) trees were constructed using the MEGA X software with a bootstrap value of 1,000 (Kumar et al., 2016). Conserved motifs were predicted for each E-class protein sequence using the MEME tool (http://meme-suite.org/), with the maximum number of motifs set to 10 (Bailey et al., 2015). Duplicates were eliminated, and all other parameters were set to default values. The conserved domains of the E-class proteins in common wheat and its ancestral/closely related species were identified using the NCBI-CDD (https://www.ncbi.nlm.nih.gov/cdd/) database (Lu et al., 2020; Mistry et al., 2021), and the results of motif and domain analyses were visualized using TBtools software (Chen et al., 2021).

### 2.3 Promoter analysis and miRNA prediction

The 2,000 base pair (bp) genomic DNA sequences upstream of the TaE transcription start sites were obtained from the common wheat reference genome IWGSC_v1.1 in the PlantCare database (http://bioinformatice.psb.ugent.be/webtools/plantcare/) (Lescot et al., 2002). They were used to identify *cis*-regulatory elements. cDNA sequences of the TaEs were used as candidate targets for predicting potential miRNAs. Using the psRNATarget Server (https://www.zhaolab.org/psRNATarget/) with default parameters, we then searched for candidate targets by screening the mature sequences of common wheat miRNAs obtained from the miRbase database (http://www.mirbase.org/) (Dai et al., 2018). The linkage of predicted miRNAs with corresponding target genes was displayed by Cytoscape software (Otasek et al., 2019).

### 2.4 Chromosomal distribution and collinearity analysis

Using the genome annotation files, we applied the Basic Circos program in TBtools to localize and visualize the identified E-class genes on chromosomes in the common wheat genome. The homology of the E-class genes in common wheat and its related wild species was then analyzed using the MCScanX and Dual Synteny Plotter tools (https://github.com/CJ-Chen/TBtools).

### 2.5 TaE expression and protein–protein interaction network analyses

To gain insight into the spatiotemporal expression patterns of the identified TaEs, we downloaded the expression data of the target genes in five wheat tissues (roots, stems, leaves, spikes, and kernels) at different stages of spike development from the WheatOmics (http://202.194.139.32/) database (Li et al., 2018; Ma et al., 2021; Pfeifer et al., 2014). Differentially expressed genes were identified based on the fragments per kilobase of transcript per million mapped reads values for each TaE, and expression levels were visualized by constructing a heatmap using the pheatmap R package. Then, the interaction networks were identified based on orthologous genes in wheat and *Arabidopsis* using the AraNetV2 (http://www.inetbio.org/aranet/) and STRING (http://string-db.org/cgi) databases, and the predicted interaction network was displayed with Cytoscape.

### 2.6 Cloning of target genes and transformation of *Arabidopsis*

Target gene sequences for PCR amplification were obtained from the Ensembl Plant database (https://plants.ensembl.org/index.html), and gene-specific primers were designed using Oligo 7 software (https://www.oligo.net/). The PCR reactions were carried out in a reaction mixture (20 μL) containing 10 μL of 2× Phanta Max MasterMix (Vazyme, Nanjing, China), 1.5 μL of cDNA, 0.5 μL each of forward and reverse primers, and 7.5 μL of enzyme-free water. The PCR amplification protocol consisted of an initial denaturation step at 98°C for 30 s, followed by 34 cycles of denaturation at 98°C for 10 s, annealing at 60°C for 20 s, and extension at 68°C for 1 min. A final extension step was performed at 68°C for 7 min. The resulting PCR products were separated on a 0.75% agarose gel by electrophoresis at 100 V for 30 min. The desired target bands were excised from the gel. The recovered PCR products were then ligated into the pMD™18-T vector (TaKaRa Bio, Kusatsu, Japan). The ligated products were subjected to restriction digestion with XbaIand KpnI(NEB, Massachusetts, American) and ligated into the overexpression vector pCAMBIA1300 (Abcam, Cambridge, UK). The recombinant vectors carrying target genes were transformed into *Agrobacterium tumefaciens* GV3101 cells (COOLABER, Beijing, China). The wild-type (WT) *Arabidopsis* Columbia ecotype and the *AT3G61120* (*TaSEP5-A* homolog) mutant *Arabidopsis* (SALK_203944C) were used as the transformation recipients. *Arabidopsis* seedlings were cultivated under at 24°C with a 16-h light and 8-h dark photoperiod. Upon maturity, seeds were collected from transgenic *Arabidopsis* plants as the T0 generation and grown on half-strength Murashige and Skoog medium containing 50 µg/mL hygromycin. Subsequently, plants were transferred to the soil. T1 generation seeds were harvested and similarly cultivated until T3 generation seeds were obtained. The genetic function of the transgenic plants was verified using the T3 generation *Arabidopsis*. The WT *Arabidopsis* transformed T3 (WT-OE), WT, mutant (MT), and transformed mutant T3 (MT-OE) seeds were cultivated in the soil. Three plantings were performed at a weekly interval. The number of days to flowering and days to maturation in these lines were determined. The morphology of fully blooming *Arabidopsis* flowers was observed using a stereomicroscope (SZX7; Olympus, Tokyo, Japan).

### 2.7 Yeast two-hybrid assay

The cloning vectors for *WLHS1-A*, *TaSEP3-A*, *TaSEP6-A*, *TaSEP4-A*, and *TaSEP4-B* were constructed using the same methods used to construct the cloning vectors for the target genes. Gene *TaSEP5-A* was ligated into the bait vector (pGADT7), whereas *WLHS1-A, TaSEP3-A, TaSEP6-A, TaSEP4-A*, and *TaSEP4-B* were ligated separately into respective prey vectors (pGBKT7). To ensure the accuracy of the experiment, we also generated several control vectors. The pGBKT-53 and pGADT7 vectors were used to co-transform yeast as positive controls. Combinations of constructed bait and blank prey vectors, as well as constructed prey and blank bait vectors, were used as negative controls. Assays involved pairing each co-transforming yeast with different combinations of each constructed bait and prey vector. The transformed yeasts were cultured on double-dropout (SD/-His-Leu) and quadruple-dropout (SD/-His-Leu-Tre-Lys) media. Protein–protein interactions (PPI) were determined based on the growth status of the yeast. The primers used for amplification are shown in **Supplementary Table S1**.

## 3 Results

### 3.1 Identification of the E-class genes in common wheat and its relatives

A total of 15 E-class genes were identified in common wheat by BLASTP. Three (*WLHS1-A*, *WLHS1-B*, and *WLHS1-D*), six (*TaSEP3-A*, *TaSEP3-B*, *TaSEP3-D*, *TaSEP5-A*, *TaSEP5-B*, and *TaSEP5-D*), and six (*TaSEP6-A*, *TaSEP6-B*, *TaSEP6-D*, *TaSEP4-A*, *TaSEP4-B*, and *TaSEP4-D*) genes were located on homoeologous group chromosomes 4, 5, and 7, respectively (**Table 1**). Using the same method, one (*AtSEP3*), one (*TuSEP3*), two (*TdSEP3-A* and *TdSEP3-B*), two (*TtSEP3-A*and *TtSEP3-B*), and three (*TsSEP3-A*, *TsSEP3-B*, and *TsSEP3-D*) E-class genes were identified in *A. tauschii* (DD), *T. urartu* (AA), *T. dicoccoides* (AABB), *T. turgidum* (AABB), and *T. spelta* (AABBDD), respectively (**Table 1**). We considered species names and chromosomal location for gene nomenclature.

**Table 1:** Nomenclature and characteristics of the putative E-class proteins in wheat and its ancestral species.

Num	Gene ID	Sequence ID	Protein size(aa)	Mw (KDa)	PI	Instability index	AI	GRAVY
1	*TuSEP3*	*TuG1812G0500003205.01*	184	21.29	9.76	52.42	84.78	-0.807
2	*AtSEP3*	*AET5Gv20667800*	287	32.87	9.34	65.46	75.85	-0.798
3	*TdSEP3-A*	*TRITD5Av1G168010*	252	29.09	9.05	56.18	76.67	-0.8
4	*TdSEP3-B*	*TRITD5Bv1G159820*	179	20.68	7.74	63.67	73.02	-0.977
5	*TtSEP3-A*	*TRIDC5AG043000*	230	26.36	9.97	53.17	72.57	-0.775
6	*TtSEP3-B*	*TRIDC5BG048600*	418	43.77	5.14	37.9	68.52	-0.42
7	*TsSEP3-A*	*TraesTSP5A01G300600*	252	29.04	8.99	56.63	76.67	-0.809
8	*TsSEP3-B*	*TraesTSP5B01G312600*	249	28.72	9	54.47	74.46	-0.837
9	*TsSEP3-D*	*TraesTSP5D01G317400*	252	29.04	8.99	56.63	76.67	-0.809
10	*WLHS1-A*	*TraesCS4A02G058900*	151	17.45	7.7	64.8	62.56	-0.686
11	*WLHS1-B*	*TraesCS4B02G245700*	156	24.95	7.61	54.59	79.86	-0.695
12	*WLHS1-D*	*TraesCS4D02G243700*	216	17.53	5.23	48.29	71.66	-0.993
13	*TaSEP3-A*	*TraesCS5A02G286800*	227	29.04	8.99	56.63	76.67	-0.809
14	*TaSEP3-B*	*TraesCS5B02G286100*	227	29.05	9	54.92	76.27	-0.809
15	*TaSEP3-D*	*TraesCS5D02G294500*	227	29.04	8.99	56.63	76.67	-0.809
16	*TaSEP5-A*	*TraesCS5A02G391800*	236	27.48	8.8	60.72	82.35	-0.645
17	*TaSEP5-B*	*TraesCS5B02G396700*	236	27.29	7.71	56.57	82.63	-0.615
18	*TaSEP5-D*	*TraesCS5D02G401700*	238	27.21	8.38	54.84	79.75	-0.634
19	*TaSEP6-A*	*TraesCS7A02G122000*	246	26.38	8.21	57.01	75.59	-0.844
20	*TaSEP6-B*	*TraesCS7B02G020800*	246	26.20	8.75	55.19	75.64	-0.806
21	*TaSEP6-D*	*TraesCS7D02G120500*	248	26.20	8.9	58.33	80.79	-0.741
22	*TaSEP4-A*	*TraesCS7A02G260600*	252	28.49	8.74	52.43	75.28	-0.814
23	*TaSEP4-B*	*TraesCS7B02G158600*	252	28.49	8.74	51.65	75.28	-0.814
24	*TaSEP4-D*	*TraesCS7D02G261600*	252	28.74	8.74	54.09	73.1	-0.82

Ta, *Triticum aestivum* (common wheat); Td, *T. dicoccoides*; At, *Aegilops tauschii*; Tu, *T. urartu*; Tt, *T. turgidum*; Ts, *T. spelta*; Mw, molecular weight; PI, isoelectric point; AI, instability index; GRAVY, grand average of hydropathicity.

The characteristics of the 24 E-class genes in six wheat species are shown in **Table 1**. The coding amino acid lengths of these genes in common wheat ranged from 151–252, with a mean size of 227 amino acids, and the molecular weights ranged from 17.5–29.1 kDa. Among the genes in the progenitors/wild relatives, the coding amino acid lengths ranged from 179–418, with a mean size of 247 amino acids, and the molecular weights ranged from 20.1–43.8 kDa. The predicted pI values of TaE proteins, ranging from 5.23–9.00 (mean: 8.30), differed significantly from those of progenitors/wild relatives (5.14–9.97, mean: 8.77). In contrast, no significant difference was found between the II (mean: 55.78), AI (mean: 76.27), or GRAVY (mean: -0.7689) values of common wheat and those of its progenitors/wild relatives (II mean: 55.03; AI mean: 75.75; GRAVY mean: −0.7862). The negative GRAVY score obtained for all E-class genes indicates that the encoded proteins are hydrophilic in nature. Furthermore, prediction of subcellular localization revealed that all the E-class genes are expressed in the nucleus.

### 3.2 Phylogenetic, motif, and domain analyses of the E-class family genes

**Figure 1:**
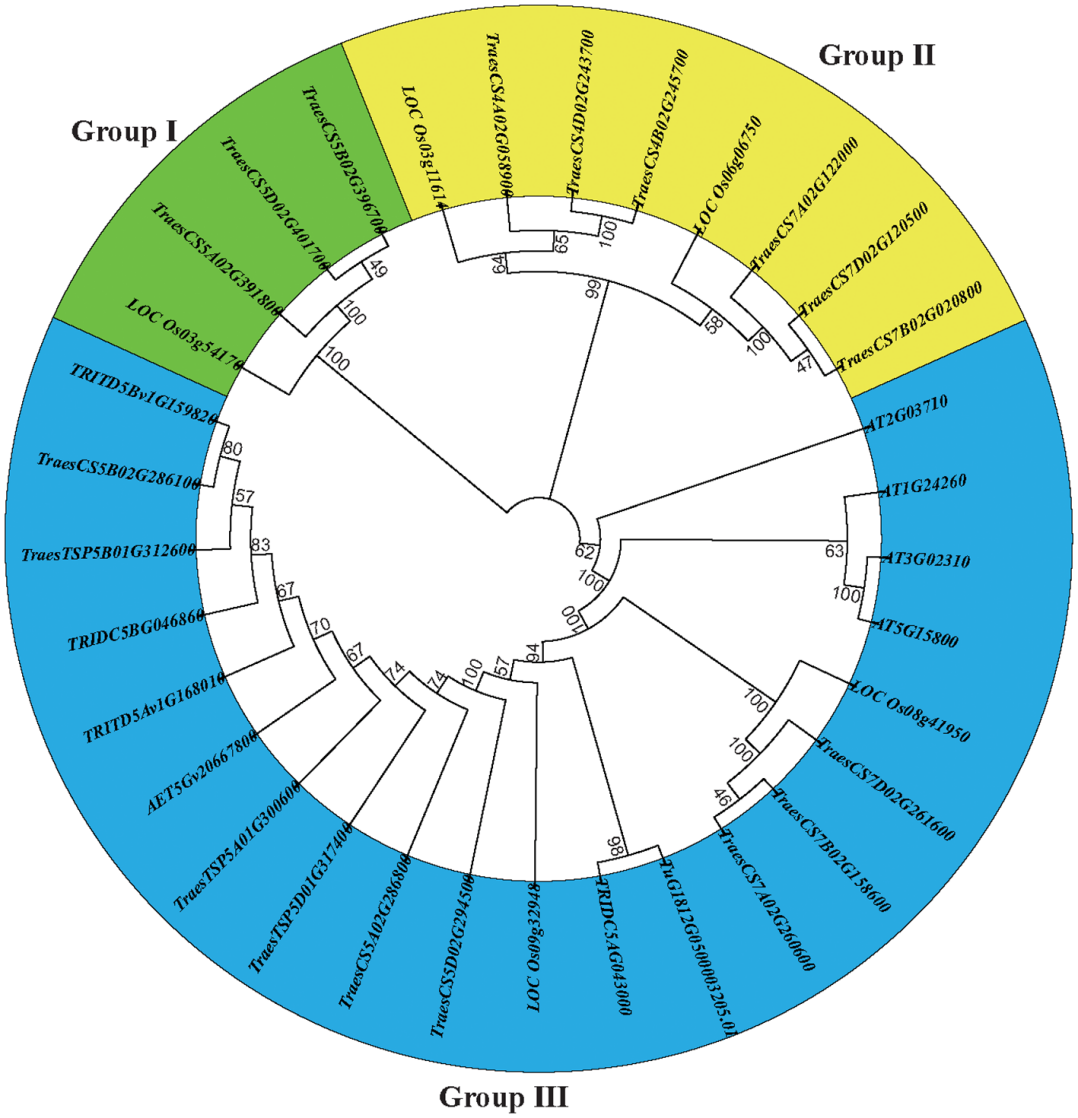
** **A dendrogram of the E-class genes constructed based on the neighbor-joining method using MEGA X with 1000 bootstrap replications.

We constructed a neighbor-joining tree to examine the phylogenetic relationships and evolutionary patterns of the E-class genes in common wheat and its ancestors. The E-class genes were categorized into three subgroups (**Figure 1**), among which subgroup I contained four genes (3 TaEs and 1 OsE), subgroup II, eight (6 TaEs and 2 OsEs), and subgroup III, 20. All the E-class genes in the progenitors/wild relatives of common wheat were placed in subgroup III, indicating that there is no significant genetic divergence among these genes.

**Figure 2:**
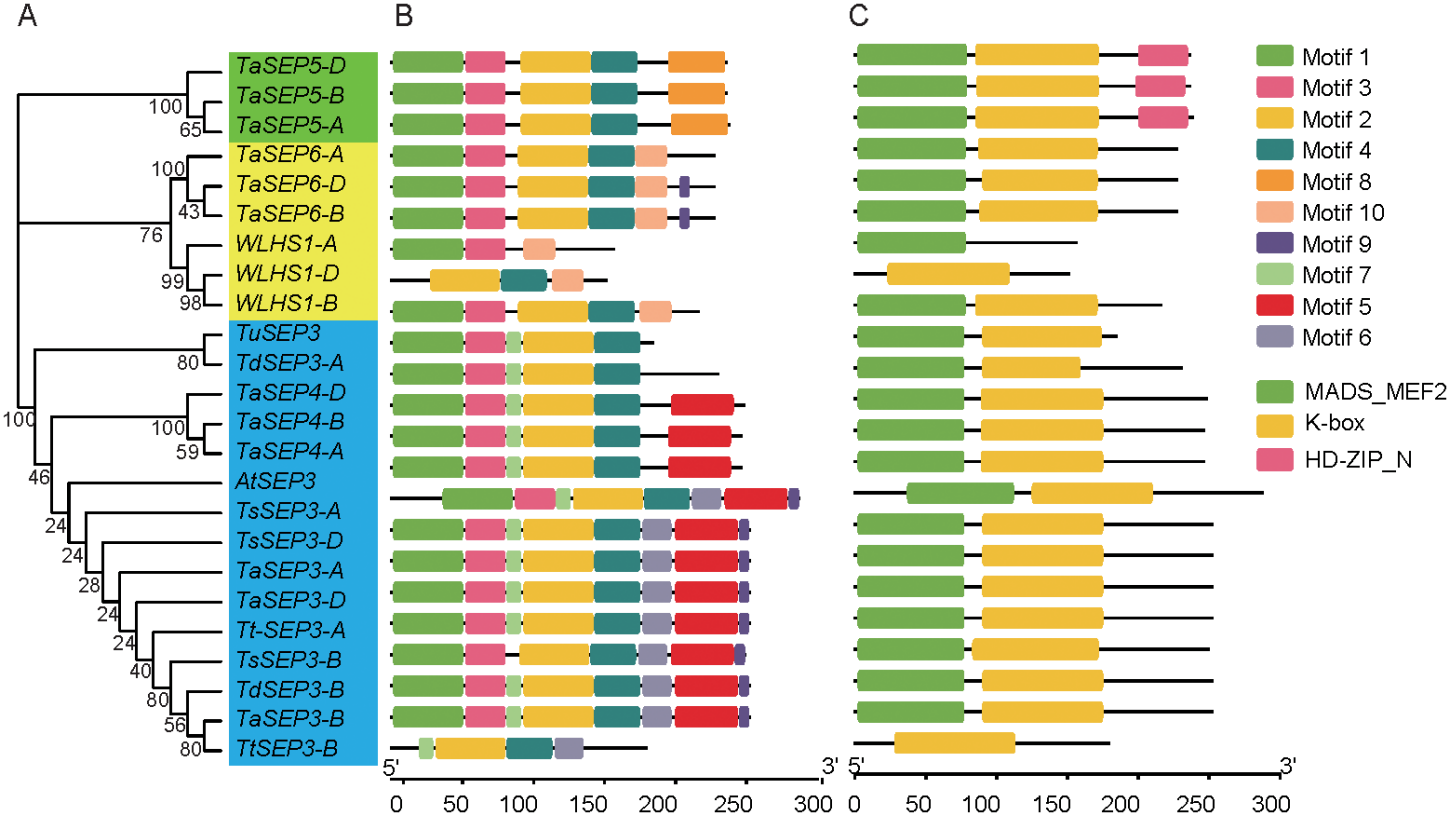
A dendrogram of the E-class genes, conserved motifs, and gene structure domains of the E-class genes in wheat and its ancestral species. **(A)** A dendrogram constructed based on the full-length amino acid sequence of the E-class genes in wheat and its ancestral species. **(B)** The conserved motifs of the E-class genes based on their phylogenetic relationship were identified using the MEME software. **(C)** The 10 types of motifs are denoted by different colors. The green, yellow, and pink rectangles represent the domains.

Based on an analysis of motifs in the 24 E-class genes from the six wheat species (**Figure 2A**), we identified a total of 10 motif types. Motifs 3 and 4 were present in 23 (all but *WLHS1-A*), and motifs 1 and 2 in 22 E-class genes. Motif 8 was specific to subgroup I. All genes in subgroup I had five motifs. Motif 10 was specific to subgroup II, with the number of motifs ranging from 3 to 6. Motif 7 was specific to subgroup III. Motif 5 was detected in 12 genes. Motifs 6 and 9 were present in 9 genes each within subgroup III (**Figure 2B**). The E-class genes in different subgroups vary in conserved motif distributions, which might suggest a conserved function of different subfamilies. Three types of conserved domains (MADS-MEF2, K-Box, and HD-ZIP_N) were identified in the six wheat species. K-Box domains were distributed in 23 E-class genes (all but *WLHS1-A*), MADS-MEF2 in 22 (all but *WLHS1-D* and *Td-5A-E1*), and HD-ZIP_N in 33 genes in subgroup I (**Figure 2C**). Most homologous genes were characterized by similar motif and domain compositions, indicating that these genes probably do not differ significantly with respect to sequence or function.

### 3.3 Chromosomal distribution of E-class genes in the six wheat species

**Figure 3:**
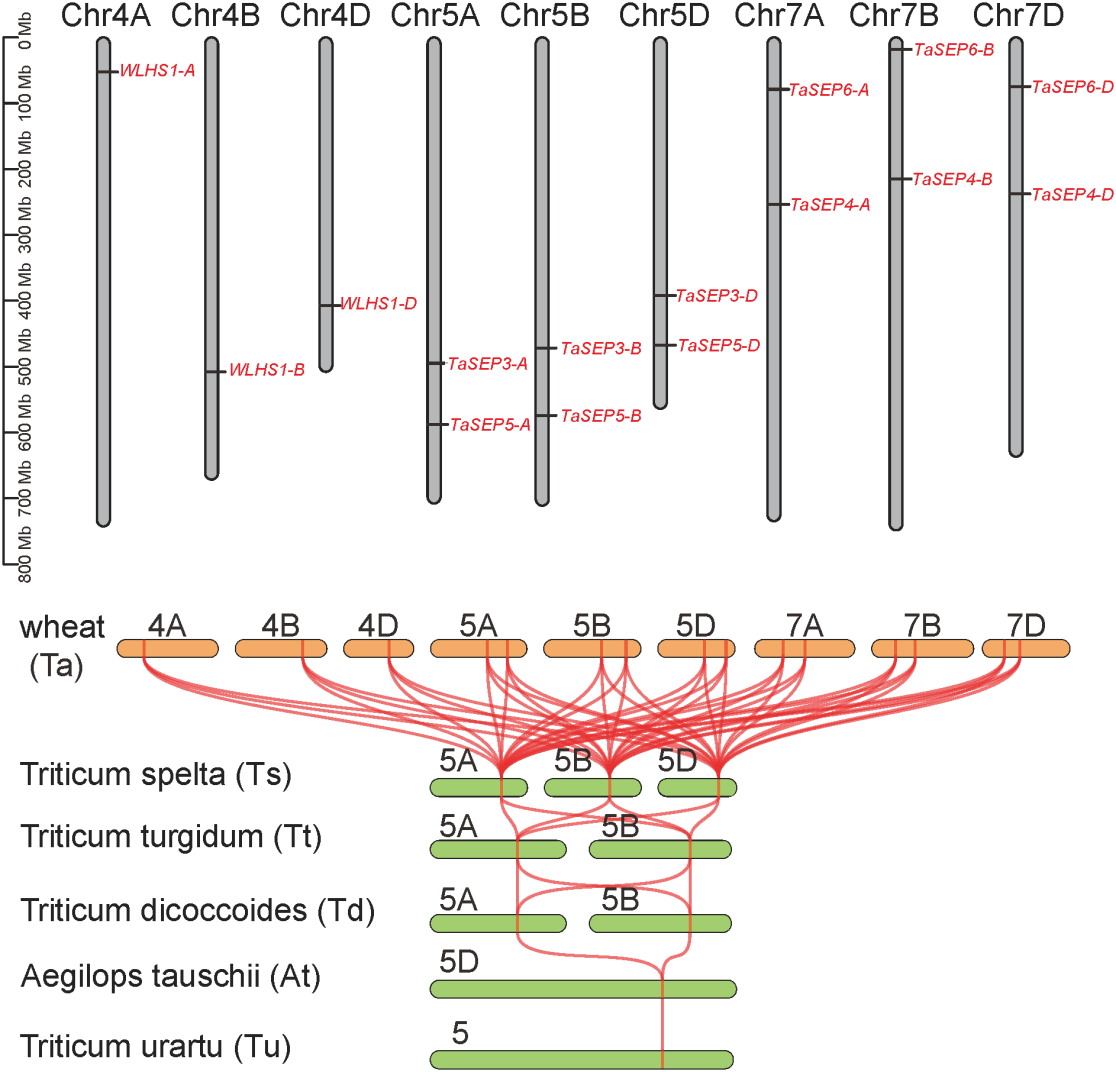
Evolution of the E-class genes in common wheat. Chromosome localization of E-class genes in the wheat genome. Collinearity analysis of the E-class genes in wheat and its ancestral species. Tu, *Triticum urartu*; At, *Aegilops tauschii*; Td, *T. dicoccoides*; Tt, *T. turgidum*; Ts, *T. spelta*; Ta, *T. aestivum*.

The TaEs were located on nine chromosomes of the homoeologous group 4, 5, and 7. The TaEs number of A, B and D subgenomes were all five, with one or two on each chromosome (**Figure 3**). The TaEs on 5 and 7 chromosomes are homologous copies of those on the wheat subgenomes A, B, and D, whereas the TaEs on chromosome 4 are not.In this regard, the specific retention or loss of homologous copies may represent evolutionary signatures of chromosomal interactions and functional divergence. The E*-*class genes were only located on group 5 chromosomes in the progenitors and wild relatives of common wheat. However, synteny analysis illustrated that all the E-class genes in the six wheat species had collinear relationships (**Figure 3**).

Molecular selection effects were assessed by comparing the dN/dS values of homologous E-class genes. No PSGs were detected in any of the groups (**Supplementary Table S1**). A total of 20, 15, and 11 NSGs were found in the wheat subgenome A (TaA) *vs.* its ancestral/closely related species subgenomes A (Tu-A-E, Ts-A-E, Td-A-E, and Tt-A-E), wheat subgenome B (TaB) *vs.* its ancestral/closely related species subgenomes B (Ts-B-E, Td-B-E, and Tt-B-E), as well as wheat subgenome D (TaD) vs. its ancestral/closely related species subgenomes D (At-D-E, Ts-D-E, Td-D-E, and Tt-D-E). The dN/dS values of Ta-E-A vs. Ta-E-B, Ta-E-A vs. Ta-E-D, and Ta-E-B vs. Ta-E-D were 0.3344, 0.3606, and 0.1915, respectively, and their NSGs are all 5. These results suggest that the E-class gene family have underwent purifying selective pressure during evolution and is rather conservative.

### 3.4 Cis-acting regulatory element analysis of TaEs

**Figure 4:**
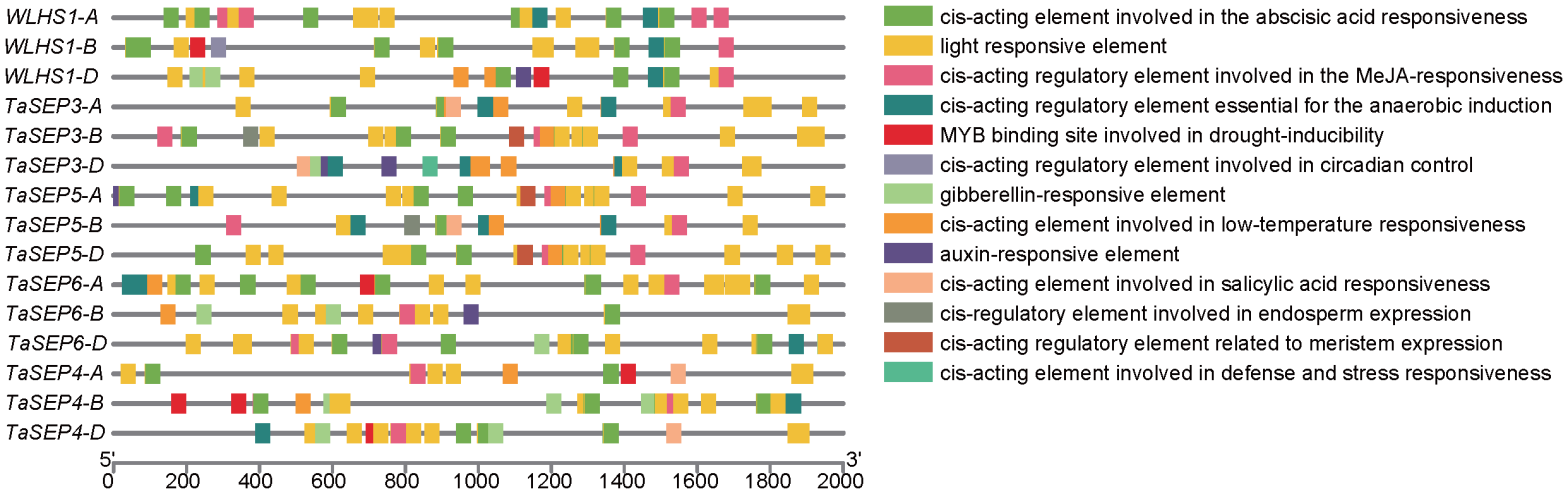
Chromosomal distribution of the TaE genes and *cis*-element analysis of the TaEs. Each E-class gene was mapped to its chromosomal location based on its physical position in the wheat genome. The chromosome number is labeled at the top of each chromosome. The scale bar is in mega bases (Mb). Different colors represent the 13 types of functional modules.

To better understand the transcriptional regulation and potential functions of TaEs, we predicted the *cis*-elements of the promoter regions for these genes and thereby identified 10 classes of functional *cis*-elements (**Supplementary Table S3**, **Figure 4**). The light-responsive element (G-Box), which is closely associated with the function of the class-E genes as key regulators of flower development, was detected in all the E-class genes. In total, 45 hormone-responsive elements were identified in these promoter regions, including 14 abscisic acid-responsive elements, 12 jasmonic acid-responsive elements (e.g., CGTCA and TGACG), nine auxin-responsive elements (AuxRR-core), five gibberellin-responsive elements (TATC), and five salicylic acid-responsive elements (TCA). These findings revealed that TaEs contain at least two hormone-related elements in their promoter regions, suggesting their extensive involvement in hormone-mediated metabolic processes and signal transduction networks in different growth and developmental processes of common wheat. We also detected stress-related elements, such as low-temperature-responsive elements; MYB binding sites involved in drought induction, defense, and stress response (TC-rich repeats); wound response elements (WUN-motif); elements associated with plant growth, such as those regulating plant meristem (CAT-box) and endosperm (GCN4) growth; and circadian elements. These findings indicate that the class-E genes play important roles not only in responding to biotic and abiotic stresses but also in the development and growth of common wheat.

### 3.5 Expression and functional analysis of TaE in *Arabidopsis*

**Figure 5:**
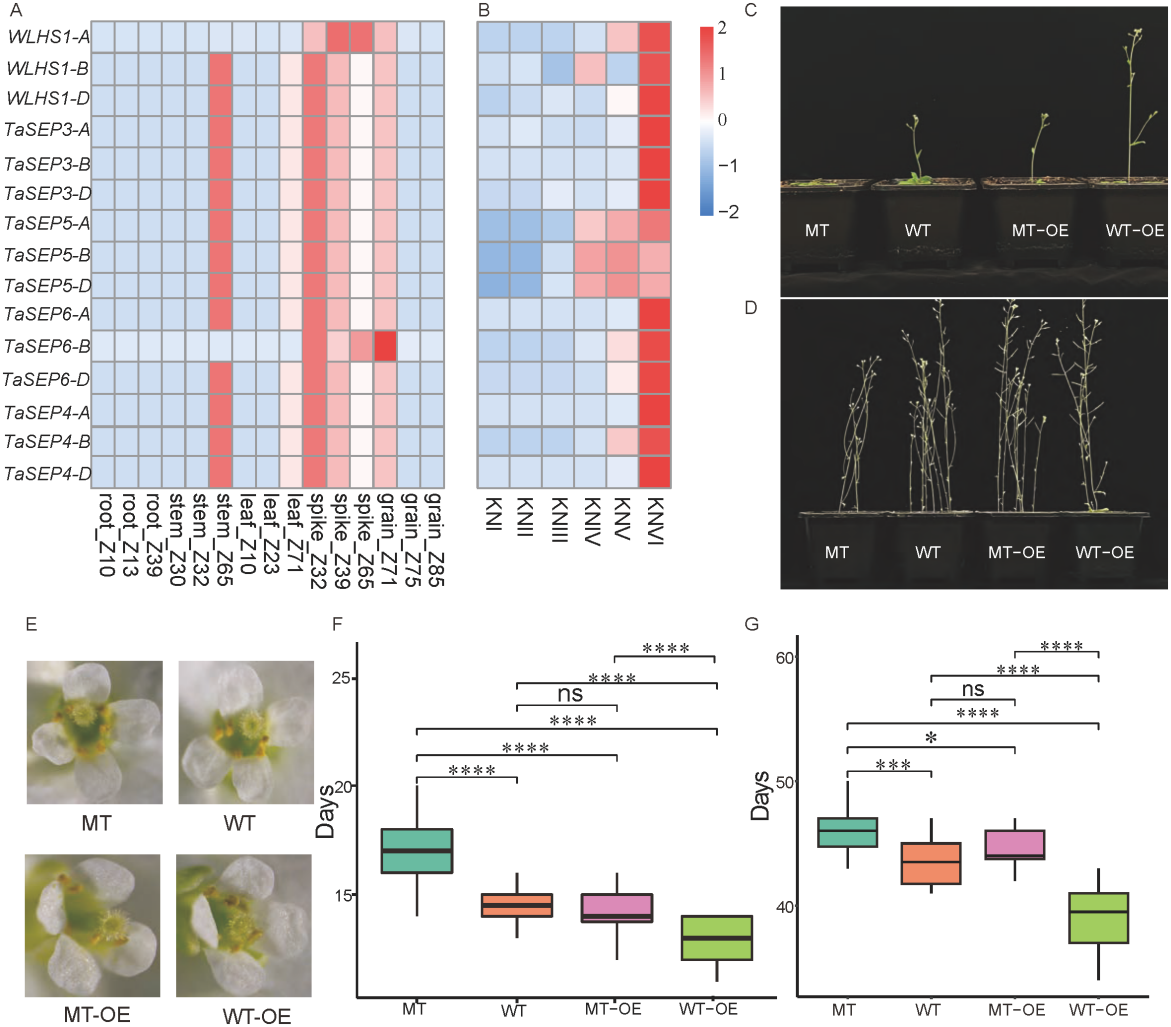
The expression levels of 15 selected TaEs, E-class genes in different parts of wheat, and E-class genes in *Arabidopsis thaliana*. **(A)** Expression of the E-class genes in different parts of wheat. **(B)** Expression of the E-class genes at different stages of wheat spike development. KNI, vegetative stage; KNII, elongation stage; KNIII, single-ridge stage; KNIV, double-ridge stage; KNV, glume primordium differentiation stage; and KNVI, floret differentiation stage. **(C)** The flowering status (single planting) of four *Arabidopsis* lines during the flowering period of the wild-type (WT) *Arabidopsis*. **(D)** The maturity status of the four *Arabidopsis* lines during the maturation of the WT *Arabidopsis*. **(E)** The morphology of flowers of the four *Arabidopsis* lines at full bloom. **(F, G)** Comparison of days to flowering and maturity in the four *Arabidopsis* lines. *, *** and **** represent significance at p < 0.05, 0.001 and 0.0001, respectively. “ns” stands for not significant (Student’s *t*-test).

Expression pattern analysis showed that all the TaEs were highly expressed in common wheat spikes (**Figure 5A**). In addition, 13 E-class genes (excluding *WLHS1-A* and *TaSEP6-B*) were highly expressed in stems. In particular, *TaSEP5-A, TaSEP5-B*, and *TaSEP5-D* were characterized by high expression from the double-ridge stage to the floret meristem stage, and the other E-class genes showed specific expression patterns in the floret meristem stage of spike development (**Figure 5B**).

To further characterize the functions and regulatory mechanisms of this gene family in common wheat, the effects of *TaSEP5-A* on spike development were investigated by transgenic and mutant (*AT3G61120*) *Arabidopsis* plants. The transgenic line was characterized by more rapid flowering. By the time that the WT *Arabidopsis* had produced its first flower, the transgenic line (WT-OE) had already produced several, the mutant line (MT) had yet to bolt, and the mutant overexpression line (MT-OE) had a phenotype like that of the WT (**Figure 5C**). The number of days to flowering for MT (16 d) was significantly longer than those for WT (13.5 d) and MT-OE plants (14 d) (*P* < 0.001), whereas that for WT-OE plants (10 d) was significantly shorter than those for MT-OE and WT plants (*P* < 0.001). No significant difference in days to flowering was detected between the MT-OE and WT plants (**Figure 5F**). Although different *Arabidopsis* lines did not vary significantly in morphology at maturity (**Figure 5D**), the days to flowering and maturity of the WT, MT-OE, MT, and WT-OE lines showed a similar trend (**Figure 5G**). Stereomicroscopic observations revealed that the different *Arabidopsis* lines were characterized by comparable flower morphologies at full bloom. We detected no evidence of developmental abnormalities or aberrations (**Figure 5E**).

### 3.6 miRNA prediction and protein–protein interactions

**Figure 6:**
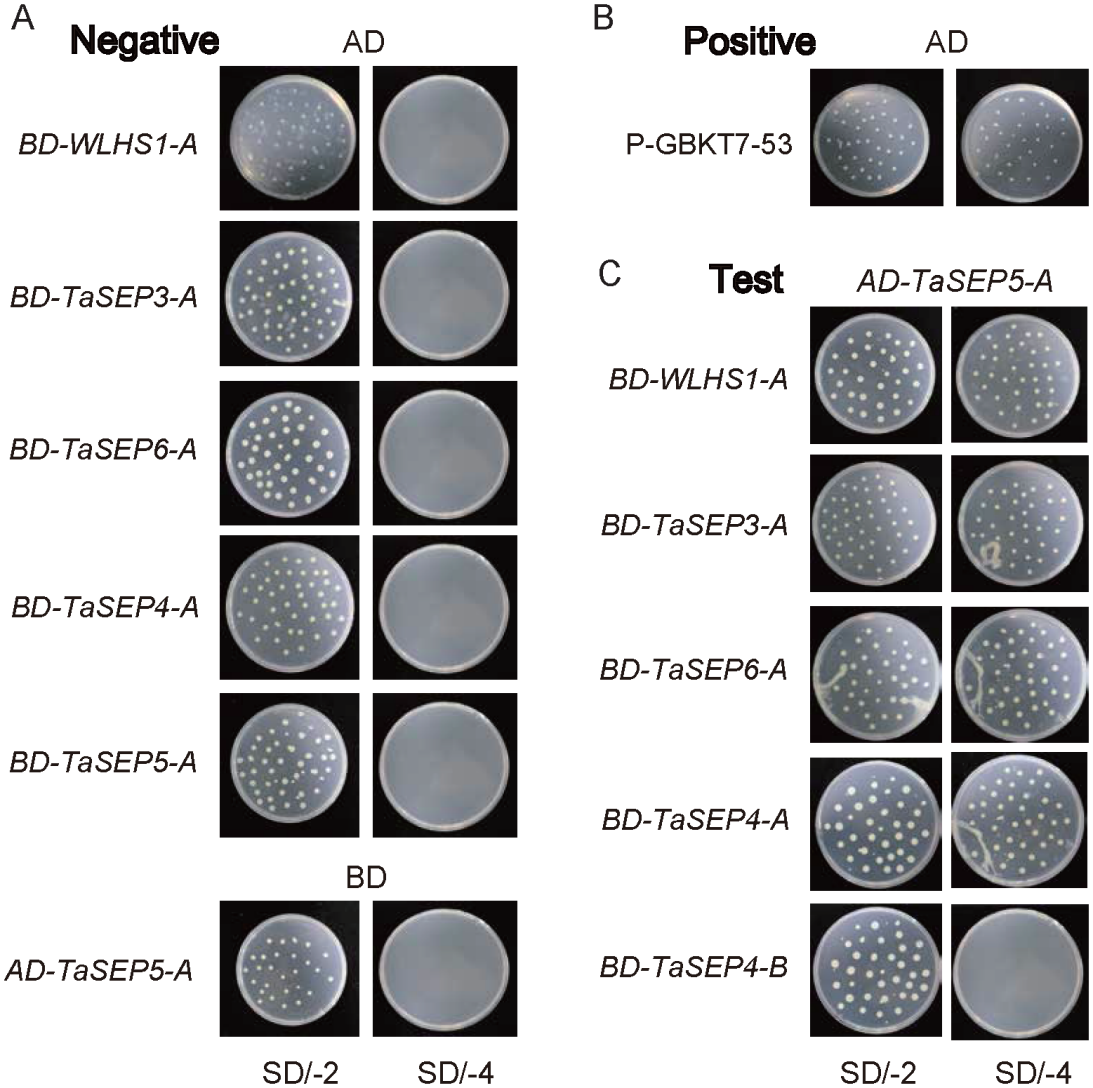
Yeast two-hybrid assay. **(A)** Negative control group. **(B)** Positive control group. **(C)** Experimental group. SD/-2: SD/-His-Leu, SD/-4: SD/-His-Leu-Tre-Lys. AD: pGADT7, BD: pGBKT7.

miRNA prediction analysis revealed four TaEs to be regulated by three miRNAs, among which two genes, *TaSEP6-B* and *TaSEP4-A*, were targeted by a single miRNA (tae-miR2275-3p), potentially a key interaction determining the effects of the E-class genes (**Supplementary Figure S1A**). According to PPI predictions, the E-class proteins are characterized by many intergroup interactions (**Supplementary Figure S1B**), as well as interactions with TaMADS, which are key transcription factors controlling flower development in wheat. Given that all the E-class genes identified in the progenitors and wild relatives of common wheat are located on chromosome 5, we selected E-class genes on chromosome 5 in common wheat for analysing intragroup interactions (**Figure 6**). Transformed yeast was cultured using double- and quadruple-dropout media. Survival status was assessed to determine whether *TaSEP5-A* interacts with other genes. Thus, we established that *TaSEP5-A* interacts with *WLHS1-A, TaSEP3-A, TaSEP6-A*, and *TaSEP4-A*, but not *TaSEP4-B*. These findings indicate that *TaSEP5-A* may have synergistic interactions with *WLHS1-A, TaSEP3-A, TaSEP6-A*, and *TaSEP4-A* in specific biological processes or signaling pathways, whereas interactions with *TaSEP4-B* may be absent or regulated by other factors.

## 4 Discussion

### 4.1 The number of homologous relationships between the E-class genes in common wheat and its progenitors and wild relatives reveal its evolutionary history

An important milestone in studying wheat evolution is the determination of the number of chromosomes in wheat (Thompson and Hake, 2009), which evidences that wheat consists of a polyploid series, including diploid (2n = 2x = 14), tetraploid (2n = 4x = 28), and hexaploid (2n = 6x = 42) wheats (Liu et al., 2021). The evolution of common wheat is assumed to have involved two rounds of autoploidization, and its genome can be arranged into seven homologous groups and three sub-genomes, with a high degree of synteny between the sub-genomes (Suárez-Baron et al., 2017).

Based on a homology comparison, 24 E-class genes were identified in the six wheat species. Phylogenetic analysis showed that the E-class genes in common wheat were distributed across all the three sub-groups, whereas those detected in the progenitors/wild relatives were found exclusively in subgroup III. Furthermore, we established that the ploidy relationship of the number of E-class genes among the progenitors/wild relatives of common wheat was consistent with the ploidy relationship of their chromosome sets. For example, the number of E-class genes in *T. dicoccoides* (AABB) was twice that of *T. urartu* (AA). These findings thus indicate that E-class genes among the progenitors/wild relatives of common wheat have a common ancestor and that the number of E-class genes accumulated with an increase in chromosome sets throughout the long-term evolution (Tsaftaris et al., 2011). During such evolution, gene duplication is the main driving force for the expansion of gene families in different species, which mainly involves segmental, tandem, and whole-genome duplication. Contrastingly, in the ancestral and closely related species of common wheat, increase in the number of E-class genes is assumed to be due to the accumulation of chromosomes over long-term evolution, rather than gene duplication (Chen et al., 2020).

The E-class genes in common wheat proved to be variously distributed on chromosomes 4 (3 genes), 5 (6 genes), and 7 (6 genes). The genes on chromosomes 5 and 7 were all homologous copies among the sub-genomes, whereas *WLHS1-A, WLHS1-B*, and *WLHS1-D* on chromosome 4 were not. This finding accordingly provides an evidence for a crossover event occurring on chromosome 4 during meiosis (Miftahudin et al., 2004). The E-class genes in the progenitors and wild relatives of common wheat were all located on chromosome 5. Thus, based on the established evolutionary process, we anticipated that the E-class genes in common wheat would similarly all be on chromosome 5 rather than distributed across multiple chromosomes (i.e., 4, 5, and 7) (Chen et al., 2020; Przewieslik-Allen et al., 2021; Zhao et al., 2021). Moreover, the number of E-class genes in cultivated wheat was markedly higher than those in its ancestral and closely related species. Taken together, these findings indicate that gene duplication occurred in response to selective pressure during the evolution of common wheat. Consistent with this scenario, the findings of previous studies have provided evidence to indicate that a large-scale structural rearrangement events occurred on chromosomes 4, 5, and 7 in common wheat (Chen et al., 2020).

In summary, by examining the evolution of E-class genes in common wheat and its ancestral/closely related species, We established the evolutionary process of common wheat and identified structural rearrangement events that occurred during this process through machine learning-based clustering tree construction and synteny analysis. These findings will provide new insights for assessing species evolution, whereby evolution can be inferred by studying the evolutionary history of a single class of key trait-related genes. Previous studies using phylogenetic analysis of key genes *TtBtr1-A* and *TtBtr1-B* clearly demonstrated that cultivated wheat has a monophyletic origin from wild wheat in the northern Levant. Further research on the newly discovered premature stop codon mutation in gene *Ppd-D1* reveals it as the first example of convergent evolution during the process of environmental adaptation in crops (Zhao et al., 2023).

### 4.2 E-class genes regulate flower spike development in common wheat

It has previously been established that the E-class genes are expressed primarily in the flowers and fruits of plants (Chahtane et al., 2023). In rice, the expression of *OsMADS7* and *OsMADS8* is limited to inflorescences and developing kernels (Cui et al., 2010; Yoshida et al., 2013). During flower development, these two genes are expressed in the floral meristems that give rise to the lodicule and stamen primordia. The expression of these genes is subsequently detected in the stamens and pistils in the lodicules until maturity (Peréz-Mesa et al., 2019). The E-class genes are highly expressed in petals of Nelumbo (Liu et al., 2021), specifically expressed in anthers and ovaries of *Aristolochia clematitis* (Peréz-Mesa et al., 2019; Yu et al., 2017), and preferentially expressed in carpels, petals, and stamens of *Carica papaya* (Lee et al., 2014). Consistently, we established that all the E-class genes in common wheat were highly expressed from the double-ridge stage to the floret meristem stage, suggesting their potential activity during these stages.

With respect to the involvement of E-class genes in flower development, knockout of the E-class genes *OsMADS7* and *OsMADS8* in rice gave rise to plants characterized by marked phenotypic variability, including late flowering, the homologous transition of calyces, stamens, and carpels to palea/lemma-like organs, and a loss of floral determinacy (Liu et al., 2013). Similarly, in response to the knockout of all E-class genes in rice, all floral organs, with the exception of lemmas, were homologously transformed into leaf-like organs in flowers (Liu et al., 2013). Similar results were observed in *Arabidopsis* (Zhao et al., 2020). Our analysis of floral development in mutant, WT, and transgenic *Arabidopsis* revealed significant differences among these lines at both anthesis and maturity. These results provide evidence that E-class genes can contribute to the regulation of flowering and maturation in *Arabidopsis*. Considering that the deletion of E-class genes can considerably influence the floral morphology in rice and *Arabidopsis*, we also performed stereomicroscopic observations of the floral morphology of different *Arabidopsis* lines during the blooming period. However, no significant changes were noted among these lines, which is consistent with the findings of previous studies (Zhao et al., 2020).

The relevance of E-class genes in regulating plant flowering processes underscores their potential application in crop improvement strategies, particularly within wheat cultivation. In-depth investigations into how these genes influence wheat floral organ development enable precise control over flowering timing and organ formation, thereby enhancing yield stability across different growing seasons and environmental conditions. Integrating these genes into wheat breeding programs facilitates the development of new varieties with enhanced stress resilience and adaptability. Amidst increasing climate variability and stress pressures, leveraging E-class genes to regulate wheat flowering and yield traits aids in breeding more robust and productive crop varieties to address global food security challenges. Elucidating the mechanisms of E-class genes in wheat provides theoretical support for developing novel genetic improvement strategies. Precision editing of these genes or introduction of superior alleles accelerates wheat breeding efforts, enhancing efficiency and success rates in genetic improvement.

### 4.3 Interactions among the E-class genes of common wheat

Through a homology-based approach, we identified many interactions among the E-class proteins of common wheat and between E-class and MADS proteins. MADS proteins regulate spike development in common wheat and thus influence overall yield (Kyoko et al., 2004). Floral homeotic MADS-domain transcription factor *SEPALLATA3* from *A. thaliana* acts as a PPI controlling flower development, because it contains leucine residues at intermolecular and intramolecular interfaces (Florian et al., 2017). This finding accordingly provides further evidence for the function of the E-class genes. In addition, we predicted the miRNAs targeting TaEs and accordingly identified four TaEs regulated by three miRNAs, with one gene targeted by two. This indicates that the TaEs are involved in a complex regulatory network. Moreover, these miRNA–TaE associations provide valuable information that could contribute to the miRNA-mediated genetic engineering of TaEs. Collectively, the integration of TaE PPI networks and miRNA-mediated pathways will contribute to a better understanding of the pathways associated with the mechanisms of action for the E-class genes in common wheat.

### 4.4 The novelty and limitations of the method

We employed a homology-based sequence comparison method, originally validated in Arabidopsis thaliana, to identify E-class genes in wheat, confirming findings consistent with prior studies (Kong et al., 2022). Additionally, we extended this method to five ancestral/closely related species, systematic analysis of E-class genes in wheat filling a critical research gap and providing new insights into the evolutionary dynamics of E-class genes in wheat using machine learning. Our comprehensive analysis also offers implications for the application of E-class genes in wheat breeding. Despite advancements in studying biological gene families, we recognize limitations in our experimental approach, specifically in functional gene exploration by transient expression in Arabidopsis rather than in wheat, which may impact accuracy and reliability. Future studies are warranted to address these challenges with more precise and in-depth analyses to validate our discoveries. Future research is necessary to validate and elucidate the functional mechanisms of E-class genes through wheat transformation experiments and multi-omics approaches. In conclusion, while acknowledging these limitations, we believe our study’s innovative perspectives and findings make significant contributions to the understanding of the wheat E-class gene family, laying a solid foundation for future research in this field.

## 5 Conclusions

Although E-class genes have been widely established to play key roles in regulating flower development in a broad range of plants, their identities and molecular functions in wheat are still poorly understood. We comprehensively analyzed the E-class genes in common wheat by using machine learning. We showed that TaE accumulation has occurred as a consequence of genome polyploidization and gene duplication during evolution. In addition, we confirmed the function of E-class genes based on expression analysis and *Arabidopsis* transformation experiments. Interacting E-class genes in common wheat were identified based on the interaction network prediction and yeast two-hybrid assays. Our findings indicate that certain TaEs have miRNA-binding sites and may accordingly be regulated by miRNAs. Although these findings provide a solid basis for understanding the potential functional roles of the E-class genes in wheat, many unresolved issues remain regarding wheat flower development. Accordingly, the potential mechanisms whereby TaEs are regulated warrant further investigation.

## Data Availability

The original contributions presented in the study are included in the article/**Supplementary Material**. Further inquiries can be directed to the corresponding authors.

## Supplementary material

Supplementary Figure 1 Gene interactions. **(A)** A schematic representation of the regulatory network relationships between the putative miRNAs and their targeted TaE genes. **(B)** Predicted protein-protein interaction networks of TaE proteins with other wheat proteins using STRING tool.
