# Supplementary figures and images for: Genome-wide identification of the E-class gene family in wheat: evolution, expression, and interaction
Source: Front Plant Sci. 2024 Sep 3;15:1419437. doi: 10.3389/fpls.2024.1419437 (PMC11405201; doi:10.3389/fpls.2024.1419437)

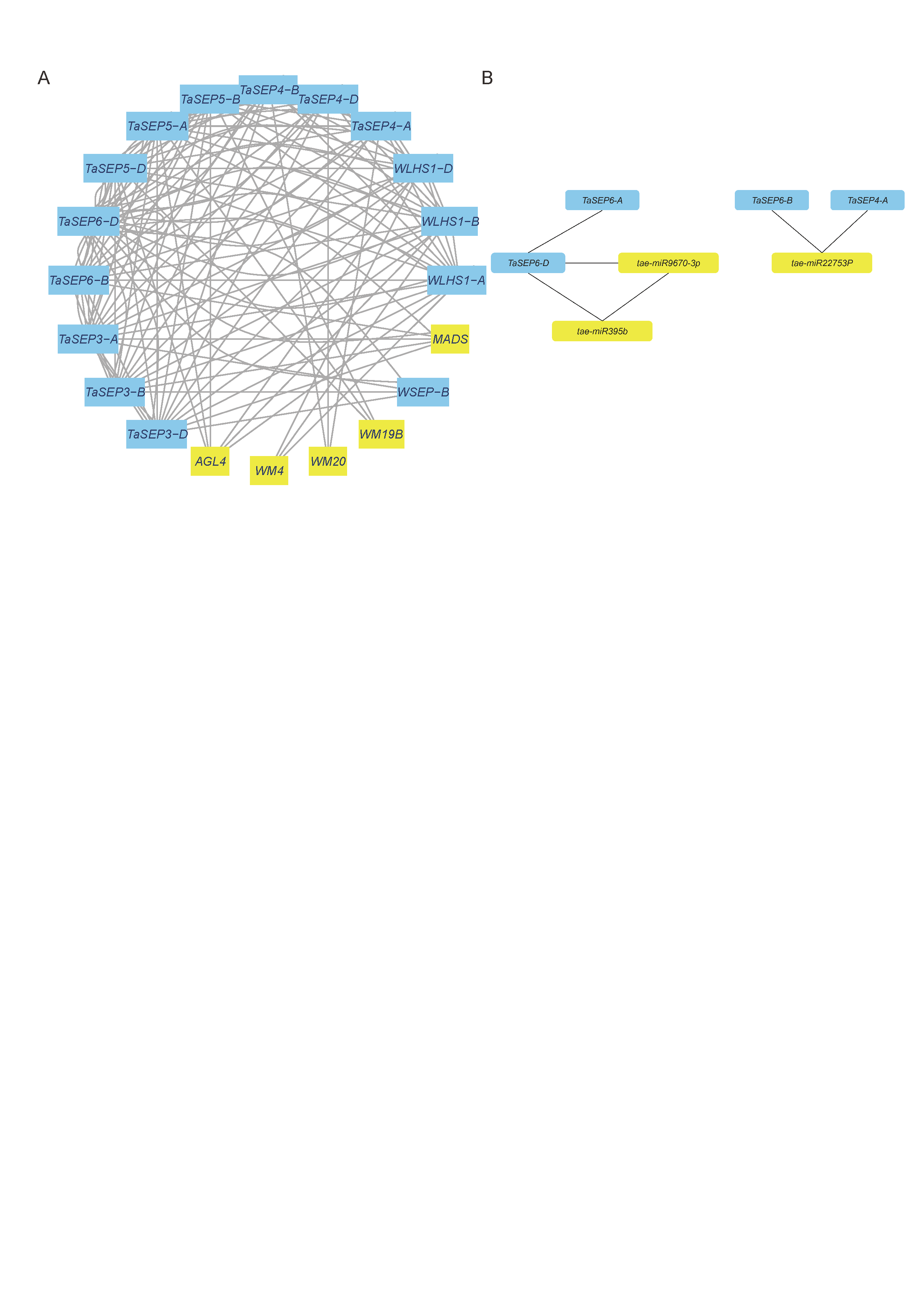

Supplement: Supplementary Figure 1 — Gene interactions. (A) A schematic representation of the regulatory network relationships between the putative miRNAs and their targeted TaE genes. (B) Predicted protein-protein interaction networks of TaE proteins with other wheat proteins using STRING tool. [file Image1.tif]
